# Impaired clinical, biological, and cytokine patterns in HIV-1 exposed uninfected newborns in Yaoundé: A comparative study of neonatal immune vulnerability

**DOI:** 10.4102/ajlm.v15i1.3035

**Published:** 2026-08-31

**Authors:** Sabine A. Touangnou-Chamda, Palmer M. Netongo, Jacky Njiki Bikoï, Véronique S. Mboua Batoum, Donatien S. Mbaga, Carole S. Sake, Marie-Christine M. Nzuno, Chris M. Nana Mbianda, Maurice Boda, Sara H. Riwom Essama

**Affiliations:** 1 Department of Microbiology, Faculty of Science, University of Yaoundé I (UYI), Yaoundé, Cameroon; 2 Molecular Diagnostics Research Group, Biotechnologic Centre, University of Yaoundé I (UYI), Yaoundé, Cameroon; 3 Department of Biochemistry, Faculty of Science, University of Yaoundé I (UYI), Yaoundé, Cameroon; 4 Biology Program, School of Science, Navajo Technical University, Crownpoint, New Mexico, United States; 5 Department of Gynaecology and Obstetrics, Yaoundé University Teaching Hospital, University of Yaoundé I (UYI), Yaoundé, Cameroon; 6 Department of Medical Laboratory Sciences, Faculty of Health Sciences, University of Buea, Buea, Cameroon; 7 Immunology and Sequencing Unit, Laboratory of Parasitology, Centre Pasteur Cameroon, Yaoundé, Cameroon; 8 Integrated Research Facility, National Institute of Allergy and Infectious Diseases, National Institute of Health, Maryland, United States

**Keywords:** human immunodeficiency virus, exposed uninfected neonates, umbilical cord blood, immune system, cytokines, Cameroon

## Abstract

**Background:**

Immune dysregulation is hypothesised to underpin the heightened susceptibility of HIV-1-exposed uninfected (HEU) infants to morbidity and mortality, which occurs at a rate three times higher than that of HIV-1-unexposed uninfected (HUU) controls. However, robust evidence substantiating this hypothesis and advocating targeted interventions remains scarce in Cameroon.

**Objective:**

To examine how maternal HIV-1 infection influences the immune response of HEU neonates in Yaoundé.

**Methods:**

A comparative cross-sectional study was conducted in 280 mother–child pairs at birth in Yaoundé. Clinical parameters were assessed, while umbilical cord blood samples from both HEU and HUU newborns were analysed for comprehensive immunophenotyping and quantification of 12 pro-inflammatory and anti-inflammatory cytokines.

**Results:**

Significant differences were observed between HEU (representing 100% of exposed newborns tested) and HUU newborns. Notably, 1/54 (1.85%) of HEU newborns exhibited a lower Appearance, Pulse, Grimace, Activity, Respiration score at 1 min (*p* = 0.01); HEU expressed severe leucocytosis (*p* = 0.008) and neutropaenia (*p* = 0.01). As well, dysregulated cytokine production was observed, with significantly elevated levels of pro-inflammatory cytokines Interleukin-6 (IL-6) (*p* = 0.003) and Interleukin-1 beta (IL-1β) (*p* = 0.004), as well as decreased levels of anti-inflammatory cytokine Transforming Growth Factor beta 1 (*p* = 0.024). Strong negative correlations were further demonstrated between monocytes and pro-inflammatory cytokines IL-1β (*r* = −0.74), Interleukin-5 (*r* = −0.75), and IL-6 (*r* = −0.75), (*p* < 0.001).

**Conclusion:**

Maternal HIV-1 infection is associated with impaired outcomes in HEU newborns, highlighted by immune dysregulation in an early pro-inflammatory environment. These findings underscore the heightened vulnerability of HEU neonates and elucidates the underlying factors contributing to their subsequent health challenges.

**What this study adds:**

It provides initial information on pertinent but not yet described pro-inflammatory cytokines impairment in Cameroonian HEU newborns. Unlike previous research that has focused on their KIR-21 gene expression and responsiveness to measles vaccine, this study introduces their immunological responses through their umbilical cord blood analysis, a previously unexamined specimen.

## Introduction

The persistent global health crisis precipitated by HIV-1 disproportionately impacts HIV-1-exposed uninfected children (HEU), constituting a significant yet often understudied population within the paediatric HIV-1 landscape.^1,2^ Remarkable advancements in preventing mother-to-child transmission through comprehensive interventions such as Option B+ implementation – which recommends a systematic and lifelong antiretroviral regimen regardless of the viral load – have demonstrably curtailed vertical HIV-1 transmission rates to approximately 2% in resource-limited settings.^3^ This success, paradoxically, has led to a substantial increase in the proportion of HEU children, now estimated at an increasing proportion globally (now reaching approximately 25% in specific high-prevalence settings in sub-Saharan Africa), with a staggering 13.2 million (90%) of the 14.8 million HEU children residing in sub-Saharan Africa.^1,4^

Emerging global clinical evidence in paediatric cohorts reveals a heightened susceptibility of HEU infants to severe infectious episodes and increased hospitalisation rates, exhibiting a threefold elevated mortality risk compared to their HIV-1-unexposed uninfected (HUU) counterparts born to HIV-1-uninfected (HU) mothers.^5,6^ Frequent hospitalisations resulting from various infectious aetiologies associated with growth retardation, have implicated immune dysfunction as a contributing factor. Mechanistically, the chronic immune activation characteristic of HIV-1-infected (HI) pregnant women, coupled with the dysregulated production of inflammatory cytokines at the maternal–foetal interface, is hypothesised to instigate aberrant inflammatory responses in the developing foetus.^7,8^

In alignment with the World Health Organization’s overarching objective to enhance the health and well-being of all children, there is an exigent imperative for health stakeholders to establish a cohesive and dedicated community focused on the specific needs of HEU children. Such a coordinated effort could help advocate for this vulnerable population, supporting their inclusion in global HIV-1 research agendas and surveillance initiatives.^9,10^

Pioneering research conducted in Cameroon has suggested that in utero exposure to HIV-1 elicits immunological dysregulation in offspring, even without vertical transmission,^11,12,13^ but strong evidence remains lacking. Subsequent foreign investigations have further delineated fundamental immunological distinctions between HEU and HUU children, including quantifiable alterations in leucocyte subset populations evident within the first months of life. Notably, an increased number of regulatory T-cells in HEU infants may contribute to diminished immune responsiveness to infections and vaccinations.^14,15^

Despite the growing body of evidence implicating foetal exposure to a chronically activated maternal immune system in shaping altered immune responses in HEU children, comprehensive published immunological profiling data for this population remain surprisingly limited within the Cameroonian context.^4,8,16^

To address this critical epidemiological knowledge gap and provide specific evidence to inform public health policy decision-makers working to establish targeted care strategies for this vulnerable group in Cameroon, the aim of this study was to examine how maternal HIV-1 infection influences the clinical status, immune cells profile, and inflammatory cytokines levels in HEU newborns compared to their unexposed counterparts in Yaoundé.

## Methods

### Ethical considerations

Prior to the collection of samples for this study, ethical clearance was obtained from the Joint Institutional Review Board for Animal & Human Bioethics (JIRB), the Ethics Committee of the University of Yaoundé I, located at the Biotechnological Centre, under reference number BTC-JIRB2022-021. All participants provided oral and/or written informed consent and signed the consent form. Blood samples were appropriately labelled, and the collected data were stored in strict compliance with confidentiality requirements according to the principles of the Helsinki Declaration. All the hospitals delivered administrative authorisations to carry out the research.^17^

### Study design and participants

**FIGURE 1:**
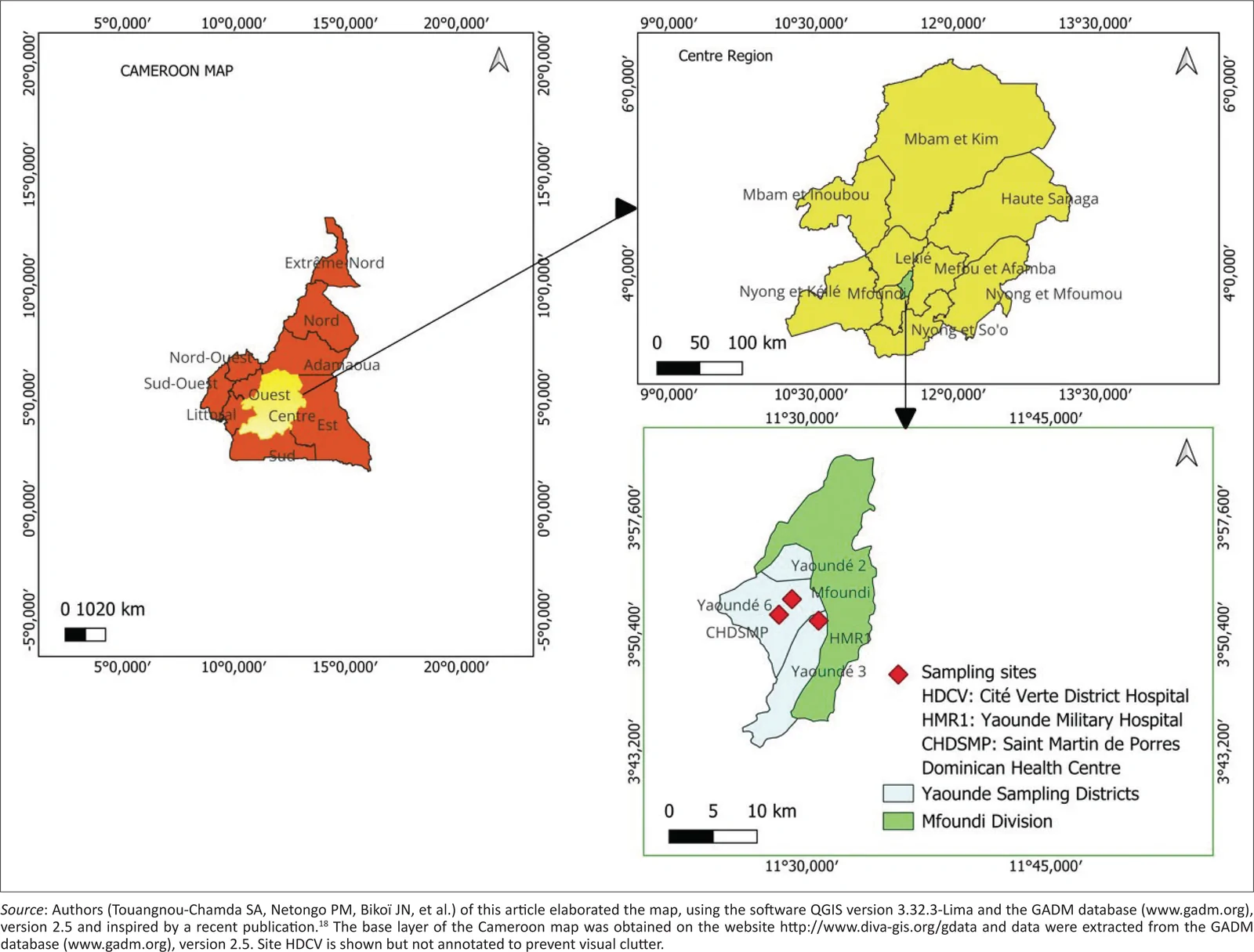
Illustrative map showing the sampling health facilities in Yaounde, Mfoundi Division, Centre Region of Cameroon from March 2022 to July 2023. The sampling sites (the Cité Verte District Hospital, Saint Martin de Porrès Dominican Health Centre, and the Yaoundé Military Hospital) are private and public health facilities where persons living with HIV are regularly followed up, under the supervision of the Cameroon Ministry of Public Health.

This was a comparative cross-sectional study with sampling conducted from March 2022 to July 2023. Sample sites were Gynaecology and Obstetrics services of three health facilities in Yaoundé, namely: the Cité Verte District Hospital, Saint Martin de Porrès Dominican Health Centre, and the Yaoundé Military Hospital (Figure 1). These facilities operate permanent Prevention of Mother-to-Child Transmission of HIV-1 programmes under the Option B+ strategy within their care units.

Sampling was consecutive and non-probabilistic. The study population comprised four groups: two groups of HI and sensitively age-matched HU mothers, as well as two groups of HEU and HUU newborns.

### Inclusion and exclusion criteria

Inclusion criteria for the exposed population were: (1) HIV-1-infected consenting delivering women of all ages (Online Supplementary Figure 1); (2) regularly followed up in the Prevention of Mother-to-Child Transmission of HIV-1 programme; and (3) presenting an undetectable viral load, along with their newborns. Their exclusion criteria included co-infection with Hepatitis B virus, Hepatitis C virus, and/or *Plasmodium* spp., to reduce immunological bias.

Inclusion criteria for the unexposed population were: (1) HIV-1-negative consenting delivering women admitted in the same hospitals as the HI women, matched by age and gestational age; and (2) control participants had no apparent cause of immunosuppression, no known chronic conditions (e.g. hypertension, diabetes, obesity, autoimmune diseases), no common viral infections, and no history of repeated blood transfusions. A detailed synoptic diagram outlining the selection criteria, sampling, and analysis conditions is provided in Online Supplementary Figure 2.

**FIGURE 2:**
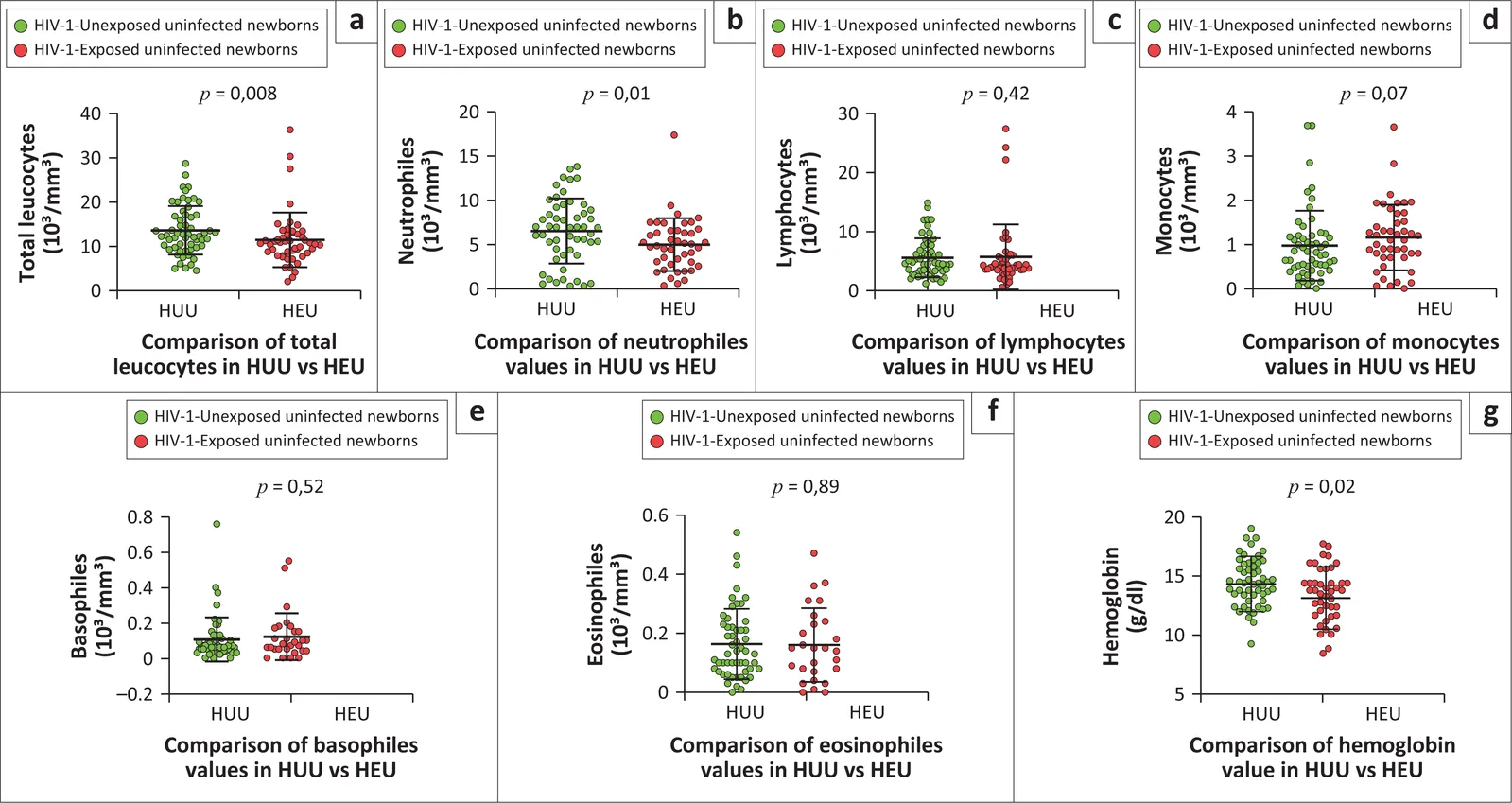
Blood count differences between HIV-1-unexposed and HIV-1-exposed newborns at HMR1, CHDSMP and HDCV hospitals in Yaoundé, Cameroon, March 2022 – July 2023. The plots illustrate measured values for individual blood cell types. Median cells values plotted on the y-axis with error bars representing one standard error from the medians. This is a detailed point-by-point comparison of Total (a) leucocytes, (b) Neutrophils, (c) Lymphocytes, (d) Monocytes and (d) Basophils, (f) Eosinophils counts, and (g) Haemoglobin level. A *p*-value less than 0.05 was used to define statistical significance. Significantly lower values of total leucocytes, neutrophils, and haemoglobin were observed in HEU newborns compared to their unexposed counterparts (*p* = 0.008 [2.a], and *p* = 0.01 [2.b], *p* = 0.02 [2.g]).

### Sample collection and processing

Studied samples included maternal venous blood and umbilical cord blood (UCB), collected in EDTA anticoagulant tubes. Peripheral blood from the heel of exposed newborns born to HI mothers was spotted onto Whatman 903 absorbent paper (GE Healthcare Life Sciences, Cytiva, Marlborough, Massachusetts, United States) and preserved as dried blood spots (Online Supplementary Figure 2). Neonatal vitality was assessed using Appearance, Pulse, Grimace, Activity, Respiration (APGAR) scores at 1, 5, and 10 min, interpreted according to established thresholds: 7–10 (normal), 4–6 (weak), and 0–3 (critical, requiring immediate medical intervention).^19,20^ Birth weight and head circumference were measured. Dried blood spot samples enabled early infant HIV-1 diagnosis. Venous blood supported full blood and differential counts, while plasma was stored at –40 °C for cytokine profiling. Analyses were performed at the Molecular Diagnostics Research Laboratory and the Immunology and Sequencing Unit, Centre Pasteur Cameroon.

### Laboratory analysis

#### HIV-1 early infant diagnosis to determine the proportion of HIV-1-exposed, uninfected newborns

Dried blood spot samples from exposed newborns were screened for HIV-1 RNA using the Abbott m2000 Real Time HIV-1 Qualitative assay (Abbott m2000rt/m2000sp, Abbott Molecular Inc., Des Plaines, Illinois, United States) via real-time polymerase chain reaction, as previously described.^21^ Umbilical cord blood samples from newborns testing negative for HIV-1 RNA polymerase chain reaction (classified as HEU) were then used for cellular profiling and cytokine quantification.

#### Cellular immune profiling

The cellular immune profile of UCB and maternal venous whole blood samples was determined using the DYMIND DF55 instrument (Dymind Biotechnology, Shenzhen, China), an automated system employing a hybrid technology involving semiconductor laser scattering, flow cytometry, and electrical impedance for blood cell enumeration. Neonatal-specific reference ranges (leucocytes: 1–30 × 10^9^/L) were used to contextualise the findings. In addition to the automated leucocyte formula, the manual May-Grünwald Giemsa technique, employing eosin and methylene blue stains, was used for differential microscopical leucocyte count.^22^

#### Plasma C-reactive protein and cytokine measurement

A panel of 12 cytokines was assayed on 88 plasma samples (22 samples from HI mothers, 22 from HU mothers, 22 from HEU newborns, and 22 from HUU newborns) according to the Clinical and Laboratory Standards Institute requirements. A representative subsample of 88 plasma samples (22 per group) was selected based on resource availability for this exploratory biomarker analysis. This panel included pro-inflammatory (Interleukin-1 beta [IL-1β], Interleukin-2 [IL-2], Interleukin-5 [IL-5], Interleukin-6 [IL-6], Interleukin-12 p70 [IL-12p70], Interleukin-18 [IL-18], Tumor Necrosis Factor alpha [TNF-α]), anti-inflammatory (Interleukin-4 [IL-4], Interleukin-13 [IL-13], Transforming Growth Factor beta 1 [TGF-β1]), antiviral (Interferon-gamma [IFN-γ]), and Granulocyte-Macrophage Colony-Stimulating Factor (GM-CSF) markers.^23^

Concentrations of TGF-β1 were determined using a simplex enzyme-linked immunosorbent assay kit (Human TGF β1 ELISA Kit No. MAN0016624, Thermofisher Scientific, Waltham, Massachusetts, United States), after pretreatment with HCl and NaOH. The absorbance was measured at 450 nm length on a spectrophotometer. The remaining 11 cytokines were quantified using the ProcartaPlex™ Human Combinable Panels MAN0028348 kit, based on Luminex xMAP technology (Thermofisher Scientific, Waltham, Massachusetts, United States). Plates were read on a Luminex MAGPix analyzer (xMAP Technology, Austin, Texas, United States), with results expressed as median fluorescence intensity.^24^

The same samples were tested for C-reactive protein, using a latex agglutination test (Biolabo SAS, Les Hautes Rives 02160, Maizy, France).^25^

### Data analysis

Data were recorded in Microsoft Excel 2016, with qualitative and quantitative variables collected for each mother–newborn pair. Statistical analyses were performed using R version 4.1.3 and GraphPad Prism version 8.4.3. Normality of quantitative distributions was assessed with Shapiro–Wilk and Kolmogorov–Smirnov tests. For normally distributed variables, means were compared using Student’s *t*-test, while non-normal data (haemogram and cytokines) were analysed with Mann–Whitney and Kruskal–Wallis tests. Cytokine values were additionally screened for outliers and reported as medians with interquartile ranges. To minimise Type I errors from multiple comparisons in cytokine data, a Bonferroni correction was applied. Spearman’s rho assessed correlations between cytokine profiles and leucocyte production in HEU newborns. Significance was set at *p* < 0.05.

## Results

A total of 280 biological samples were obtained from 140 mother–child pairs, comprising 65 pairs of HI mothers and their exposed neonates, alongside 75 pairs of HU mothers and their unexposed neonates.

### Differences and similarities observed between HIV-1-infected and HIV-1-uninfected mothers regarding their sociodemographic and clinical parameters

A comparative analysis of sociodemographic and clinical parameters between HU (*N* = 75) and HI (*N* = 65) mothers was undertaken (Table 1). Educational attainment differed significantly (*p* = 0.007), with a markedly higher proportion of HI mothers (81.8%) limited to primary education, whereas HU mothers were predominantly represented in higher education (70.6%). Age also varied significantly between groups (*p* = 0.0002), though standard deviations overlapped. In contrast, maternal weight (75 kg), gestational age (39 weeks), systolic and diastolic blood pressure, and marital status revealed no statistically significant differences (*p* > 0.05), thereby indicating comparability across these parameters and underscoring overall homogeneity in clinical characteristics (Table 1).

**TABLE 1:** Comparative analysis of sociodemographic and clinical data on HIV-1 infected and uninfected mothers selected at three referral hospitals in Yaoundé, Cameroon, March 2022 to July 2023.

Clinical parameters	HIV-1 uninfected (HU) (*N* = 75)						HIV-1 infected (HI) (*N* = 65)						*p*
	Mean	Median	s.d.	IQR	*n*	%	Mean	Median	s.d.	IQR	*n*	%	
Age (years)	27.47	-	5.82	-	-	-	31.14	-	5.40	-	-	-	0.0002*
Weight (kg)	-	75	-	69–85	-	-	-	75.5	-	68.25–82.75	-	-	0.99
Gestational Aage (WA)	-	39.6	-	39–40.40	-	-	-	39.55	-	37.30–40.40	-	-	0.29
Systolic BP (mmHg)	121	-	11.50	-	-	-	119.5	-	±14.17	-	-	-	0.22
Diastolic BP (mmHg)	-	80.5	-	70–100	-	-	-	78.5	-	70–87	-	-	0.28
**Sociodemographics**													0.007*
Education level													
Primary School	-	-	-	-	2	18.2	-	-	-	-	9	81.8	
High School	-	-	-	-	17	48.6	-	-	-	-	18	51.4	
Higher Education	-	-	-	-	24	70.6	-	-	-	-	10	29.4	
**Marital status**													0.78
Single	-	-	-	-	28	57.14	-	-	-	-	21	42.86	
Cohabiting (Free union)	-	-	-	-	7	58.33	-	-	-	-	5	41.67	
Married	-	-	-	-	17	65.38	-	-	-	-	9	34.62	
**HIV clinical history**													
Time of diagnosis													
During current pregnancy	-	-	-	-	NA	NA	-	-	-	-	7	11.86	NA
Before current pregnancy	-	-	-	-	NA	NA	-	-	-	-	52	88.14	NA
**ART regimen**													
TELE	-	-	-	-	NA	NA	-	-	-	-	36	55.38	NA
TLD	-	-	-	-	NA	NA	-	-	-	-	29	44.62	NA

Note: Under strong selection criteria (HI mothers under ART with undetectable viral load and HIV uninfected mothers with no immunodeficiency evidence), only a few significant differences were found between the demographic data of HU and HI mothers. Missing data are not considered in the Table 1.

s.d., standard deviation; IQR, interquartile range; ART, antiretroviral treatment; TELE, Tenofovir + Lamivudine + Efavirenz; TLD, Tenofovir + Lamivudine + Dolutegravir; WA, weeks of amenorrhoea; NA, not applicable.

* , A *p* value less than 0.05 was considered statistically significant.

### Clinical difference in HIV-1-exposed uninfected children and HIV-1-unexposed uninfected newborns, with notable significance in appearance, pulse, grimace, activity, respiration vitality score (*p* = 0.01)

A comparative analysis of demographic and clinical characteristics between HUU and HEU newborns was conducted (Table 2). The sex ratio differed between groups (0.96 for HEU vs 0.6 for HUU), although no significant association between HIV-1 exposure and infant gender was found (*p* = 0.16). Clinical parameters revealed notable trends. Mean birth weight (3289 ± 426.5 g for HUU vs 3281 ± 484.2 g for HEU) and head circumference (median 35 cm vs 34 cm) showed no significant differences (*p* = 0.92 and *p* = 0.09). However, HEU infants included a minimum birth weight of 2000 g, below the 2500 g threshold. Importantly, a significant difference emerged in APGAR scores at 1 min (*p* = 0.01) (Table 2).

**TABLE 2:** Comparison of demographic and clinical data of HIV-1-unexposed, uninfected and HIV-exposed, uninfected newborns at three referral hospitals (HMR1, CHDSMP and HDCV) in Yaoundé, Cameroon, March 2022 to July 2023.

Variables	HIV-1 Unexposed uninfected (HUU)						HIV-1 Exposed uninfected (HEU)						*p*
	Number	s.d.	%	IQR	Min	Max	Number	s.d.	%	IQR	Min	Max	
**Gender**													0.16
Women	42	-	62.7	-	-	-	28	-	51.0	-	-	-	
Men	25	-	37.3	-	-	-	27	-	49.0	-	-	-	
**Way of delivery**					-								0.36
Normal	67	-	93.0	-	-	-	46	-	87.0	-	-	-	
Caesarian section	5	-	7.0	-	-	-	7	-	13.0	-	-	-	
**Weight (g)**	3289	± 426.5	-	-	2520	4500	3281	± 484.2	-	-	2000	4550	0.92
**Head circumference (cm)**	35	-	-	34–36	32	39	34	-	-	33–35.5	31	37	0.09
**Weak APGAR score (4–6) at 1 min**	0/63	-	0.0	-	7	10	1/54	-	1.9	-	6	10	0.01

Note: Missing data were not considered in the Table 2. A *p*-value less than 0.05 was considered statistically significant.

s.d., standard deviation; IQR, interquartile range; APGAR, appearance, pulse, grimace, activity, respiration.

### Full proportion of exposed uninfected newborns born from undetectable viral load mothers

String selection criteria were considered in the HI women cohort recruitment, illustrated in a flowchart. Out of 102 HI women initially approached, 65 HI mothers with undetectable viral load were considered, and 65 dried blood spot samples from their newborns were tested for early infant diagnosis of HIV-1. Results revealed that 100% of HIV-1-exposed newborns were uninfected, or HEU (Online Supplementary Document 1).

### White blood cells and haemoglobin trends in HI and HU mothers

A comparative evaluation of haematological parameters, including haemoglobin levels and leucocyte subsets, was performed between HU and HI mothers, all of whom exhibited values within the normal physiological range. Globally, no statistically significant difference was observed (*p* = 0.92), reflecting overall homogeneity. However, analysis of individual indices revealed a modest yet significant variation in haemoglobin levels (*p* = 0.03), suggesting minor differences in red cell indices. Notably, median values for neutrophils, lymphocytes, and monocytes were identical – approximately 5 × 10^3^/mm^3^ (neutrophils), 1.5 × 10^3^/mm^3^ (lymphocytes), and 0.5 × 10^3^/mm^3^ (monocytes) – underscoring the remarkable similarity in these leucocyte populations between HU and HI mothers (Supplementary Table 1 and Supplementary Table 2).

### Impaired total white blood cells and neutrophils count between HIV-1-exposed uninfected children and HIV-1-unexposed uninfected newborns

A comparison between various blood cell counts and haemoglobin levels in UCB of HUU and HEU newborns is presented below. Panel 2.b illustrates that neutrophils counts were, similarly to leucocytes (2.a), significantly diminished in HEU newborns compared to HUU newborns (*p* = 0.01), highlighting a specific impairment in this critical innate immune cell population. Furthermore, Panel 3.g revealed that haemoglobin levels were significantly lower in HEU newborns compared to HUU newborns (*p* = 0.02), suggesting a compromised erythroid status in the exposed cohort. These findings collectively indicate that while certain leucocyte subsets remain comparable, HIV-1 exposure (even without viral transmission), could be associated with a reduction in total leucocytes, neutrophils, and haemoglobin levels in newborns (Figure 2).

### Comparative analysis of leucocyte counts trends across all groups

**FIGURE 3:**
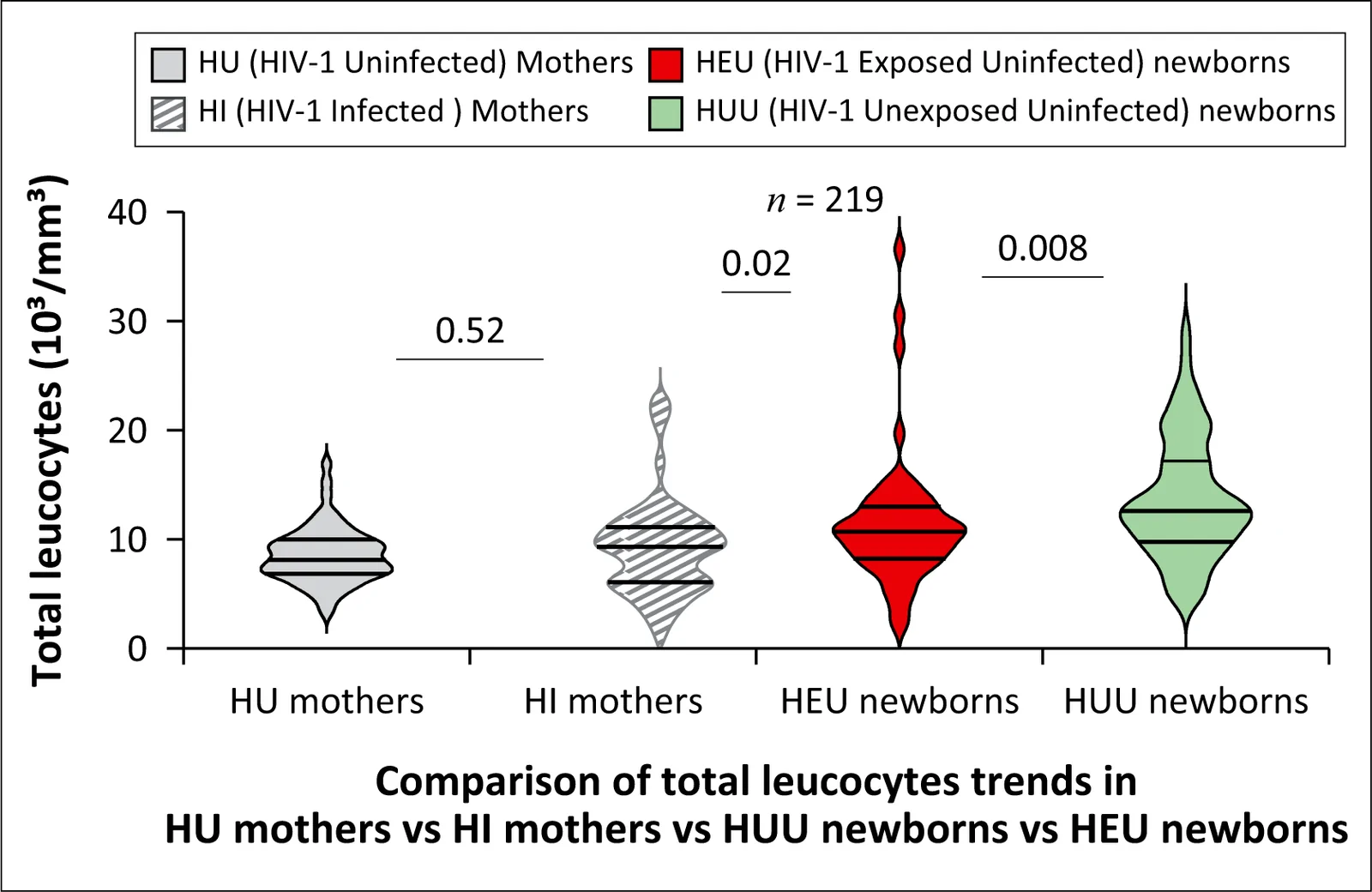
Global comparison of total leucocyte count across all groups at HMR1, CHDSMP and HDCV hospitals in Yaoundé, Cameroon, March 2022 - July 2023. Violin plots depict total leucocyte values for the four groups: HU and HI mothers; HUU and HEU newborns. Medians and interquartile ranges are visualised in three-layered distributions. This analysis highlights significant leucopaenia in HEU newborns relative to HUU newborns. Additionally, the similar distribution patterns observed between HI and HU mothers suggest relative comparability in leucocyte profiles across maternal cohorts.

A comparative analysis of total leucocyte counts across four groups – HU and HI mothers, HUU and HEU newborns – was conducted using violin plots to depict distributions and median values (Figure 3). Both HU and HI mothers exhibited relatively low, narrowly distributed leucocyte counts, with no significant difference (*p* = 0.52). In contrast, newborns displayed greater variability. HEU neonates demonstrated significantly lower median leucocyte counts and narrower distributions compared with HUU counterparts (*p* = 0.008). Furthermore, overall median white blood count values indicated that leucocyte patterns in HEU newborns more closely resembled adults (*p* = 0.02), suggesting potential maturational or developmental alterations (Figure 3).

### Almost similar cytokines quantification in HIV-1-infected and HIV-1-uninfected mothers

The cytokine profile of the study population revealed distinct patterns between mothers and newborns. In mothers, comparative analysis of the 12 tested cytokines represented in Supplementary Table 1 indicated a general homogeneity between HU and HI groups, with most cytokine medians being statistically similar; for instance, IL-2 (16.54 pg/mL, *p* = 0.54), IL-4 (26.92 pg/mL, *p* = 0.67), IL-12p70 (10.81 pg/mL, *p* = 0.76), IL-13 (27.93 pg/mL, *p* = 0.95), and GM-CSF (149.8 pg/mL, *p* > 0.99). The only statistically significant difference observed was for the anti-inflammatory cytokine TGF-β1 (*p* = 0.028), suggesting a subtle modulation in this specific pathway in infected mothers. Overall, several cytokine values in mothers, including IL-4, IL-6, IL-13, GM-CSF, IL-18, and TGF-β1, were above the 20 pg/mL threshold (Supplementary Table 2).

### Quantified upregulation in pro-inflammatory cytokines Interleukin-1 beta and Interleukin-6, and downregulation in anti-inflammatory transforming growth factor beta 1 expression in neonates’ umbilical cord blood

**FIGURE 4(a-l):**
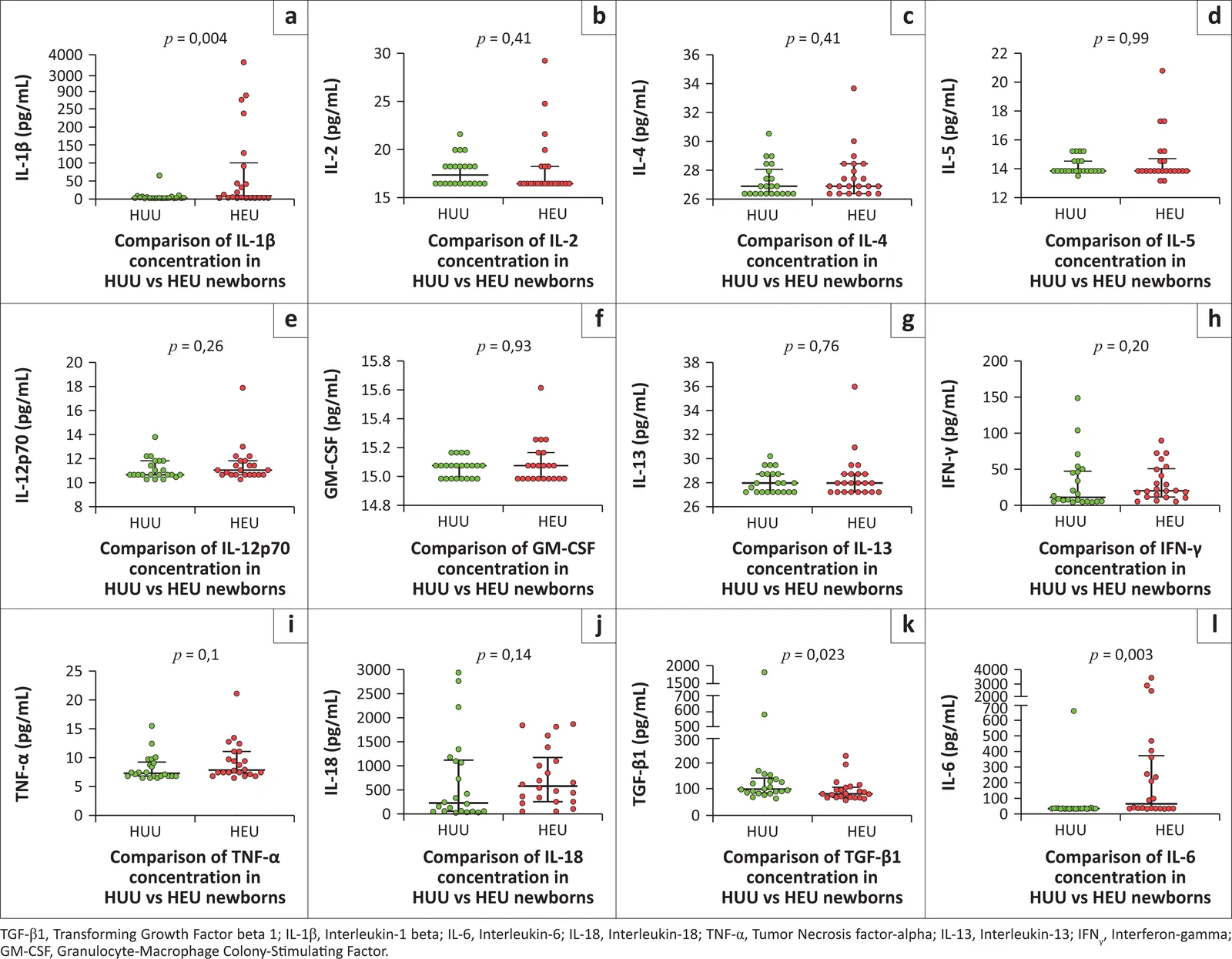
Graphical representation of cytokine values within HUU and HEU newborns, using umbilical cord blood plasma samples collected at HMR1, CHDSMP and HDCV hospitals in Yaoundé, Cameroon, March 2022 – July 2023.

In contrast to non-significantly different C-reactive protein values, while high expression (> 20 pg/mL) was noted for IL-4, IL-6, IL-13, GM-CSF, IL-18, and TGF-β1 across both HEU and HUU groups, three statistically significant differences emerged within neonates. HEU neonates’ UCB exhibited a significantly elevated expression of pro-inflammatory cytokines IL-6 (*p* = 0.003) (Figure 4), IL-1β (*p* = 0.004) (Figure 4) and a significantly reduced expression of the anti-inflammatory cytokine TGF-β1 (*p* = 0.003) (Figure 4). Furthermore, although not statistically significant, HEU neonates also showed a strong expression of the pro-inflammatory cytokines IFN-γ (19.41 pg/mL vs 10.29 pg/mL for HUU, *p* = 0.2) and IL-18 (574.5 pg/mL vs 225.9 pg/mL for HUU, *p* = 0.14), indicating a pro-inflammatory skew in the immune profile of HEU newborns (Figure 4, Supplementary Material 5).

The plots illustrate cytokine levels in HIV-1-unexposed, uninfected newborns (HUU) compared to their HIV-1-exposed, uninfected (HEU) counterparts. Median cytokine values are plotted on the *y*-axis, with error bars representing one standard error from the median. Statistically significant differences were observed in TGF-β1, IL-1β, and IL-6 expression levels between HEU and HUU groups, with a *p*-value < 0.05.

### Negative correlation between monocytes and pro-inflammatory cytokines in umbilical cord blood of HIV-1-exposed uninfected children newborns

The Spearman correlation was applied between the cellular and cytokine profiles of HEU newborns, and correlation coefficients (*r*) with corresponding *p*-values were represented below, highlighting statistically significant relationships (*p* < 0.05). First, total white blood cell counts exhibited a significant intermediate negative correlation with IL-1β (*r* = −0.46, *p* = 0.04). Second, monocyte counts demonstrated multiple significant negative correlations: strong negative correlations with IL-1β (*r* = −0.74, *p* < 0.001), IL-5 (*r* = −0.75, *p* < 0.001), and IL-6 (*r* = −0.75, *p* < 0.001), and intermediate negative correlations with IL-2 (*r* = −0.63, *p* = 0.004), IL-12p70 (*r* = −0.47, *p* = 0.04), GM-CSF (*r* = −0.50, *p* = 0.03), and TNF-α (*r* = −0.55, *p* = 0.01). These findings suggest that lower monocyte levels are associated with elevated levels of these pro-inflammatory and T-cell-related cytokines. Third, eosinophil counts displayed several strong positive correlations: with IL-1β a (*r* = 0.79, *p* = 0.04), IL-2 (*r* = 0.87, *p* = 0.02), IL-5 (*r* = 0.87, *p* = 0.01), and TNF-α (*r* = 0.78, *p* = 0.04), indicating that higher eosinophil counts are associated with increased levels of these specific cytokines. Notably, redundancy was observed for IL-1β with all three cell types (total white blood cells, monocytes, and eosinophils) showing significant correlations (Table 3 and Table 4).

**TABLE 3:** Correlation matrix between cellular and cytokines profile of HIV-1-exposed, uninfected newborns: *r* values at HMR1, CHDSMP and HDCV hospitals in Yaoundé, Cameroon, March 2022 - July 2023.

Cells versus cytokines	IL-1β	IL-2	IL-4	IL-5	IL-6	IL-12p70	IL-13	IFN_γ_	GM-CSF	TNF_α_	IL-18	TGF β 1
Haemoglobin	−0.12	0.40	0.07	0.25	0.17	−0.01	0.13	−0.08	0.55	0.1	−0.06	−0.50
Leucocytes	−0.46	−0.06	−0.34	−0.24	−0.22	−0.31	−0.22	−0.43	0.03	−0.27	−0.38	−0.02
Platelets	0.15	0.49	0.41	0.21	−0.07	0.38	0.43	0.24	0.30	0,.41	0.30	0.29
Neutrophils	−0.36	0.12	−0.09	−0,08	−0.20	−0.16	0.05	−0.21	0.13	−0.1	−0.10	0.14
Lymphocytes	−0.19	0.28	0.04	0.08	−0.13	0.03	0.24	−0.13	0.30	0.15	−0.06	0.05
Monocytes	−0.74	−0.63	−0.47	−0.75	−0.75	−0.47	−0.38	−0.24	−0.50	−0.55	−0.24	0.001
Eosinophils	0.79	0.87	0.47	0.87	0.78	0.65	0.44	0.21	0.78	0.78	0.21	0.14
Basophils	−0.15	−0.01	−0.44	−0.30	−0.20	−0.21	−0.43	−0.62	−0.12	−0.17	−0.62	0.19

IL-1β, Interleukin-1 beta; IL-2, Interleukin-2; IL-4, Interleukin-4; IL-5, Interleukin-5; IL-6, Interleukin-6; IL-12p70, Interleukin-12 p70; IL-13, Interleukin-13; IFN_γ_, Interferon-gamma; GM-CSF, Granulocyte-Macrophage Colony-Stimulating Factor; TNF_α_, Tumor Necrosis Factor alpha; IL-18, Interleukin-18; TGF-β1, Transforming Growth Factor beta 1.

**TABLE 4:** Correlation matrix between cellular and cytokines profile of HIV-1-exposed, uninfected newborns: *p* values at HMR1, CHDSMP and HDCV hospitals in Yaoundé, Cameroon, March 2022 – July 2023.

Cyto/Cellular	IL-1β	IL-2	IL-4	IL-5	IL-6	IL-12p70	IL-13	IFN_γ_	GM-CSF	TNF_α_	IL-18	TGF-β1
Haemoglobin	0.63	0.11	0.78	0.33	0.51	0.95	0.614	0.74	0.,02	0.70	0.82	0.042
Leucocytes	0.04	0.79	0.13	0.31	0.34	0.17	0.35	0.05	0.89	0.24	0.10	0.93
Platelets	0.57	0.04	0.09	0.41	0.76	0.12	0.08	0.33	0.24	0.09	0.27	0.24
Neutrophils	0.14	0.62	0.73	0.74	0.41	0.53	0.83	0.39	0.61	0.71	0.68	0.59
Lymphocytes	0.45	0.25	0.87	0.75	0.59	0.89	0.32	0.60	0.22	0.54	0.82	0.84
Monocytes	< 0.001	0.004	0.05	< 0.001	< 0.001	0.04	0.11	0.32	0.03	0.01	0.34	0.99
Eosinophils	0.04	0.02	0.28	0.01	0.05	0.12	0.32	0.66	0.05	0.04	0.66	0.78
Basophils	0.71	0.98	0.27	0.47	0.61	0.62	0.27	0.10	0.77	0.69	0.10	0.65

The Spearman correlation was applied between the cellular and cytokine profiles of HIV-1-exposed, uninfected newborns, and correlation coefficients (*r*, value up) with corresponding *p*-value (value down) highlighted statistically significant relationships when *p* < 0.05.

IL-1β, Interleukin-1 beta; IL-2, Interleukin-2; IL-4, Interleukin-4; IL-5, Interleukin-5; IL-6, Interleukin-6; IL-12p70, Interleukin-12 p70; IL-13, Interleukin-13; IFN_γ_, Interferon-gamma; GM-CSF, Granulocyte-Macrophage Colony-Stimulating Factor; TNF_α_, Tumor Necrosis Factor alpha; IL-18, Interleukin-18; TGF-β1, Transforming Growth Factor beta 1.

## Discussion

This study demonstrates that HEU newborns in Yaoundé exhibit distinct clinical and immunological associations compared to those born to uninfected mothers (HUU). To better assess these immunological sequelae, only HI mothers with undetectable viral load and no co-infections were included, alongside HU mothers with no signs of immunodeficiency. Therefore, cellular and soluble immune mediators (cytokines) in maternal venous blood and UCB of both groups were quantified. Subsequent correlational analyses aimed to identify relationships between these immune parameters in HEU neonates to understand the potential impact of the in-utero antiretroviral therapy (ART) exposure environment.

### Impaired neonatal clinical outcomes in HIV-1-exposed uninfected children from healthy mothers

With matching criteria for maternal enrolment, maternal weight was comparable between the two groups (mean 75 kg), emphasising general well-being in infected mothers under persons living with HIV Option B+ follow-up interventions.^26^

Significant differences observed between neonatal clinical parameters in HEU and HUU, notably lower 1-min APGAR scores, smaller mean head circumference, and a higher incidence of low birth weight suggest a potential negative impact of in utero ART exposure on early neonatal anatomy and physiology. This could be linked to maternal HIV-1 infection (even with viral suppression), chronic subclinical inflammation, or ART-associated effects on placental development and foetal growth.^27,28^ These observations align with studies in Zimbabwe and Kenya that reported ART-associated reductions in neonatal anthropometrics, including increased low birth weight in HEU infants.^6,29^

### Quantified haematological profile alterations in HIV-1-exposed uninfected children neonatal compartments

Similarly, the displayed neonatal leuconeutropaenia in HEU UCB is likely a consequence of multifactorial aetiologies, potentially involving maternal anaemia and nutritional deficiencies in HI mothers (indicated by lower haemoglobin, *p* = 0.03). Some HEU UCB exhibited lower neutrophil counts at 370 cells/mm^3^, representing a 94.7% decrease from the lower reference limit (Online Supplementary Table 3). HIV-exposed pregnancies may alter foetal haematopoiesis via ART myelosuppression, epigenetic immune programming, and subclinical inflammation. These factors may affect neutrophil dynamics through microbial transplacental transfer, cytokines, or ART-induced mitochondrial dysfunction in HEU neonates, though these remain speculative mechanisms that require further longitudinal investigation.^27,28,30,31^ The observed lower maternal haemoglobin in HI mothers, a factor known to independently affect neonatal haematology,^31,32^ supports the finding of reduced neutrophil counts in HEU infants,^7^ suggesting a potential link between complex in utero exposures and the hypothesised heightened inflammatory environment in these neonates.^33^

### Quantified dysregulation of Interleukin-1 beta, Interleukin-6 and Transforming Growth Factor beta 1 expression in HIV-1-exposed uninfected children neonates

The significant upregulation of pro-inflammatory cytokines IL-6 and IL-1β in HEU neonates is consistent with prior findings of elevated IFN-γ and IL-1β (*p* < 0.01).^34,35,36^ This supports the hypothesis that in utero exposure to ART-controlled maternal HIV infection is associated with an enhanced foetal immune response,^34^ as evidenced by significantly higher cytokine levels in the cord blood of HEU newborns compared to HUU controls. The finding of elevated IL-6, contrasting with reduced IL-22 production in a Kenyan study,^6^ is mechanistically plausible, as Th22 cell differentiation depends on TNF-α and IL-6.^6^ While the study of Denney et al.^37^ did not find significant differences in IL-6 levels but reported a decrease in IL-22, their finding corroborates the decreased TGF-β1 levels observed in this cohort, suggesting that in utero HIV exposure may reduce the production of anti-inflammatory cytokines through yet-to-be-defined mechanisms. This reduced anti-inflammatory capacity could compromise neonatal homeostasis, with an impact on foetal growth as observed in the low birth weight.^37^ This study provides evidence of elevated specific proinflammatory cytokines in HEU neonates. While the precise mechanisms remain unclear, these findings align with reports linking maternal ART to mitochondrial toxicity and metabolic disturbances, though these remain speculative mechanisms that require further longitudinal investigation.^6,34^

### Quantified inverse correlation between monocyte levels and pro-inflammatory cytokines Interleukin-1, Interleukin-5, and Interleukin-6 in HIV-1-exposed uninfected children neonates

This negative correlation could be explained by the differentiation of circulating monocytes into tissue-resident macrophages upon infiltration, leading to a decrease in circulating monocyte numbers while these macrophages secrete substantial quantities of pro-inflammatory cytokines.^38^ This finding is consistent with a prior study demonstrating a significant correlation between monocyte levels and IL-1β levels in HEU children,^39^ indicative of heightened inflammation and immune activation. Maternal ART (especially protease inhibitors) disrupts foetal mitochondrial function, enhancing IL-1β expression and suppressing monocyte differentiation via inflammatory pathways, although these remain speculative mechanisms that require further longitudinal investigation.^40^

### Limitations of the study

The stringent selection of study participants effectively mitigated potential confounding variables, thereby enabling a robust evaluation of in vivo immunological status under controlled and optimised experimental conditions in Cameroon. However, several limitations must be acknowledged. First, the cross-sectional nature of this study prevents establishing causality between maternal exposures and neonatal outcomes. Second, the cytokine analysis was limited to a subsample of the total cohort because of resource constraints. Third, the latex agglutination test for C-reactive protein analysis is a method that presents a limited sensitivity. Finally, the lack of longitudinal follow-up means the persistence of these immunological associations beyond the immediate neonatal period could not be assessed. Furthermore, while plasma cytokine quantification established immunological impairments, intracellular cytokine analysis^41^ would have offered additional insights into the functional status of the immune system.

### Conclusion

This study investigated the sequelae of maternal HIV-1 infection on the immune status of HEU newborns, indicating potential immunomodulation linked to systemic inflammation. Compared to HUU neonates, HEU newborns presented with trends towards poorer clinical parameters at birth characterised by lower birth weight, smaller head circumference, and significantly lower APGAR scores at 1 min. These were accompanied by impaired biological markers such as decreased leucocytes, neutrophils, and haemoglobin levels, coupled with elevated pro-inflammatory cytokines IL-6 and IL-1β, and downregulated TGF-β1. These findings suggest that in utero exposure to maternal HIV infection may establish a persistent inflammatory milieu in HEU newborns, potentially compromising their subsequent immune responsiveness. Further research is warranted to optimise clinical management strategies tailored to this heightened vulnerability in HEU infants.
